# *Clonorchis sinensis* lysophospholipase A upregulates IL-25 expression in macrophages as a potential pathway to liver fibrosis

**DOI:** 10.1186/s13071-017-2228-z

**Published:** 2017-06-17

**Authors:** Lina Zhou, Mengchen Shi, Lu Zhao, Zhipeng Lin, Zeli Tang, Hengchang Sun, Tingjin Chen, Zhiyue Lv, Jin Xu, Yan Huang, Xinbing Yu

**Affiliations:** 1 Department of Parasitology, Zhongshan School of Medicine, Sun Yat-sen University, Guangzhou, China; 2 Key Laboratory for Tropical Diseases Control, Sun Yat-sen University, Ministry of Education, Guangzhou, Guangdong China

**Keywords:** *Cs*LysoPLA, Liver fibrosis, IL-25

## Abstract

**Background:**

Liver fibrosis is an excessive wound-healing reaction that requires the participation of inflammatory cells and hepatic stellate cells (HSCs). The pathogenesis of liver fibrosis caused by viruses and alcohol has been well characterized, but the molecular mechanisms underlying liver fibrosis induced by the liver fluke *Clonorchis sinensis* are poorly understood. Lysophospholipase A (LysoPLA), which deacylates lysophospholipids, plays a critical role in mediating the virulence and pathogenesis of parasites and fungi; however, the roles of *C. sinensis* lysophospholipase A (*Cs*LysoPLA) in *C. sinensis*-induced liver fibrosis remain unknown.

**Methods:**

A mouse macrophage cell line (RAW264.7) was cultured and treated with *Cs*LysoPLA. IL-25 and members of its associated signaling pathway were detected by performing quantitative real-time PCR, Western blotting and immunofluorescent staining. A human hepatic stellate cell line (LX-2) was cultured and exposed to IL-25. LX-2 cell activation markers were examined *via* quantitative real-time PCR, Western blotting and immunofluorescent staining. Migration was analyzed in transwell plates.

**Results:**

Treating RAW264.7 cells with *Cs*LysoPLA significantly induced IL-25 expression. Elevated PKA, B-Raf, and ERK1/2 mRNA levels and phosphorylated B-Raf and ERK1/2 were detected in *Cs*LysoPLA-stimulated RAW264.7 cells. The PKA inhibitor H-89 weakened B-Raf and ERK1/2 phosphorylation whereas the AKT activator SC79 attenuated ERK1/2 phosphorylation in RAW264.7 cells. Both H-89 and SC79 inhibited *Cs*LysoPLA-induced IL-25 upregulation. In addition, stimulation of LX-2 cells with IL-25 upregulated the expression of mesenchymal cell markers, including α-smooth muscle actin (α-SMA) and collagen type I (Collagen-I), and promoted cell migration.

**Conclusions:**

*Cs*LysoPLA activates HSCs by upregulating IL-25 in macrophages through the PKA-dependent B-Raf/ERK1/2 pathway and potentially promotes hepatic fibrosis during *C. sinensis* infection.

## Background

Clonorchiasis, a food-borne zoonosis, is caused by *Clonorchis sinensis* infection [1–3]. Adults of *C. sinensis* parasitize the intra-hepatic bile ducts of their hosts. Long-term infection by *C. sinensis* results in chronic liver injury leading to liver fibrosis [4, 5]. Mechanical damage caused by the adult *C. sinensis* worm and excretory/secretory proteins (ESPs) as well as the interplay between worms and the host immune system are responsible for pathological changes [6, 7]. However, the exact molecular mechanisms involved in *C. sinensis-*induced liver fibrosis remain unclear.

IL-25 (also known as IL-17E) is a member of the IL-17 cytokine family and is considered a T helper type 2 (Th2) cell-derived cytokine [8]. IL-25 is also expressed in alveolar macrophages, mast cells and eosinophils [9–11]. Unlike the proinflammatory effects exerted by other members of the IL-17 family, IL-25 promotes type 2 inflammation by locally upregulating IL-4, IL-5 and IL-13 [8, 9]. In mice, the intranasal administration or forced expression of IL-25 induces pulmonary inflammation similar to asthma [12, 13]. Administration of an IL-25 blocking antibody in allergen-exposed mice results in a moderate reduction in airway inflammation [14]. IL-25 also has the ability to modulate tumor pathogenesis. IL-25 administration in mouse xenograft models of human melanoma, breast, lung, colon, and pancreatic cancers induces antitumor activity that requires the presence of B cells and eosinophil infiltration [15]. In addition, IL-25, which is essential for host defense, is induced at high levels following helminth infection [16, 17].

Liver fibrosis is an excessive wound-healing reaction associated with chronic injury to the liver, such as that caused by virus and parasite infections, alcohol abuse, and metabolic and autoimmune diseases [18, 19]. When the liver is subjected to chronic injury, hepatic stellate cells (HSCs) are exposed to autocrine or paracrine signals, including oxidative stress, apoptotic bodies, and cytokines such as TGF-β1 and PDGF, and transform into activated myofibroblast-like cells [20]. Activated HSCs not only generate extracellular matrix (ECM) but also secrete cytokines and growth factors to promote the development of liver fibrosis [21]. Liver fibrosis is a sequela of various inflammatory processes comprising both innate and adaptive immune responses [22, 23]. Infection with *C. sinensis* is characterized by a Th2-dominant immune response, which is vital for the development of liver fibrosis [24–26], and hepatic macrophages also reportedly play a critical role [27]. Supporting this link, macrophages were shown to produce IL-25 in a rat model of particle-induced airway inflammation [9]. IL-25 is a Th2 cytokine, and according to the same study investigating rat airway inflammation, hepatic macrophages overexpress IL-25 and may contribute to liver fibrosis caused by *C. sinensis*.

Lysophospholipase A (LysoPLA) is a member of the phospholipase family and has been identified in many mammalian tissues and cells. This enzyme deacylates lysophospholipids and likely plays a pivotal role in the virulence and pathogenesis of parasites and fungi [28–30]. Previously, we expressed and characterized *C. sinensis* lysophospholipase A (*Cs*LysoPLA) and observed that it upregulated the expression of pro-fibrotic genes in a hepatic stellate cell line (LX-2) [30, 31]. In the present study, we detected IL-25 levels in a macrophage cell line (RAW264.7) treated with *Cs*LysoPLA in vitro and analyzed levels of signaling molecules. Furthermore, we evaluated cell migration and mRNA expression levels in LX-2 cells after IL-25 administration.

## Methods

### Expression and purification of recombinant *Cs*LysoPLA

As previously described [30, 31], the *Cs*LysoPLA coding region was amplified by polymerase chain reaction (PCR) using a cDNA plasmid library derived from adult *C. sinensis* worms as a template. The PCR product was cloned into the His_6_-tagged expression vector pET-28a(+) after digestion with BamH I/Xho I (Thermo Fisher Scientific, Waltham, MA, USA). The recombinant plasmid was then transformed into *Escherichia coli* BL21 (DE3) for overexpression induced by isopropyl-β-D-thiogalactoside (IPTG). *Escherichia coli* were harvested by centrifugation and resuspended in phosphate-buffered saline (PBS), sonicated on ice, and centrifuged to collect the supernatant. The recombinant protein was purified using a His Bind Purification Kit (Novagen, Darmstadt, Germany), eluted with 150 mM imidazole and dialyzed in PBS to remove the imidazole.

### Culture and treatment of RAW264.7 and LX-2 cells

RAW264.7 cells (2.5 × 10^5^ cells/well) were seeded in 24-well plates in Dulbecco’s modified Eagle’s medium (DMEM) (Gibco, Carlsbad, USA) containing 10% heat-inactivated fetal bovine serum (FBS), 100 U/ml penicillin, and 100 μg/ml streptomycin. Cells were cultured at 37 °C in an atmosphere containing 5% CO_2_ until they reached 80% confluence. The cells were then exposed to *Cs*LysoPLA (1, 5, 10 or 20 μg/ml) for 12 h, 24 h and 48 h, *C. sinensis* fructose-1,6-bisphosphatase (*Cs*FBPase) (20 μg/ml) and mouse serum albumin (MSA, 20 μg/ml) (Fitzgerald, MA, USA) as control proteins, or equal volumes of PBS as blank controls. To investigate the effects of signaling inhibitors or activators, RAW264.7 cells were treated with *Cs*LysoPLA (10 μg/ml) in the presence or absence of H-89 (a PKA inhibitor) (20 μM) (Beyotime, Shanghai, China) or SC79 (an AKT activator) (4 μg/ml) (Sigma-Aldrich, Steinheim, Germany) for 15 min, 30 min, 60 min and 90 min as previously described [32–34]. LX-2 cells (2 × 10^5^ cells/well) were seeded in 6-well plates and grown to 70% confluence at 37 °C in an atmosphere containing 5% CO_2_ followed by serum starvation for 24 h. The cells were then stimulated with IL-25 (20 ng/ml) (R&D, Minneapolis, USA) with or without BAY 11–7083 (an NF-κB inhibitor) (0.1 μg/ml) (Beyotime, Shanghai, China) for 24 h; TGF-β1 (5 ng/ml) (Peprotech, Rocky Hill, USA) was used as a positive control.

### Reverse transcription and quantitative real-time PCR

Total cellular RNA was extracted from RAW264.7 or LX-2 cells using TRIzol reagent according to the manufacturer’s protocol. cDNA was synthesized using a RevertAid First Strand cDNA Synthesis Kit (Thermo Fisher Scientific, Waltham, MA, USA) and amplified on a Bio-Rad CFX96 Real-Time system (Bio-Rad, Hercules, CA, USA) with SYBR Green I (Takara, Dalian, China) and specific primers for quantitative analysis. Briefly, the cDNA was pre-denatured at 95 °C for 30 s, followed by 40 cycles at 95 °C for 5 s and 60 °C for 30 s. β-actin was amplified as a house-keeping gene for each sample. Relative fold-changes in mRNA expression were determined by calculating 2^-ΔΔCt^. Primer sequences are listed in Table 1.

**Table 1:** Primer sequences for quantitative real-time PCR

Gene	Sequence (5′-3′)	Accession number
α-SMA	Forward: CCAGGGCTGTTTTCCCATCC	NM_001613.2
	Reverse: GCTCTGTGCTTCGTCACCCA	
IL-25	Forward: TCTACCGAGTCTCCTTGGCT	NM_080729.3
	Reverse: ATTGTACACCTGGCCCTCTC	
TNF-α	Forward: GACAGTGACCTGGACTGTGG	NM_013693.3
	Reverse: TGAGACAGAGGCAACCTGAC	
iNOS	Forward: ACCTTGTTCAGCTACGCCTT	NM_010927.3
	Reverse: CATTCCCAAATGTGCTTGTC	
IL-6	Forward: AGTCCGGAGAGGAGACTTCA	NM_031168.1
	Reverse: ATTTCCACGATTTCCCAGAG	
PKA	Forward: CCTGTTCCCACCCTATCACT	NM_001277898.1
	Reverse: TGGAAGCCATCACTCAGTCT	
B-Raf	Forward: TCCACGTTGGCATTGTTAGT	XM_006505358.2
	Reverse: TCACTCCTGTAAGCGTCCTG	
ERK1	Forward: TCCCAGGAGGACCTTAATTG	NM_011952.2
	Reverse: AAGGTTAACATCCGGTCCAG	
ERK2	Forward: TGAGGATGTTAGGCTTCGTCT	NM_001038663.1
	Reverse: AAAGTCCACTCCCACAATGC	
β-actin^a^	Forward: TGGACTTCGAGCAAGAGATG	NM_001101.3
	Reverse: GAAGGAAGGCTGGAAGAGTG	
β-actin^b^	Forward: GGAATGGGTCAGAAGGACTC	NM_007393.5
	Reverse: CATGTCGTCCCAGTTGGTAA	

^a^ *Homo sapiens*

^b^ *Mus musculus*

### Immunofluorescent staining

RAW264.7 or LX-2 cells were cultured on slides. The cells were fixed with 4% paraformaldehyde for 20 min at room temperature (RT), then washed with PBS and permeabilized in PBS containing 0.3% Triton X-100 for 10 min. The slides were blocked with PBS containing 1% bovine serum albumin for 30 min and incubated with a primary monoclonal antibody against IL-25 (R&D, Minneapolis, USA) or collagen type I (Collagen-I) (Abcam, London, UK) overnight at 4 °C. The slides were then incubated with a Cy3-conjugated or FITC-conjugated secondary antibody (Proteintech, Chicago, USA) for 1 h in darkness. Finally, the slides were stained with 4′,6-diamidino-2-phenylindole (DAPI) and mounted with antifade reagent (Beyotime, Shanghai, China). Images were obtained using an Olympus BX63 microscope and cellSens Dimension (Version1.8) software (Olympus, Tokyo, Japan). The intensity of IL-25 and collagen type I staining was analyzed with Image-Pro Plus v6.0 software.

### Western blotting

Protein lysates from cells receiving different treatments were prepared using RIPA buffer (Beyotime, Shanghai, China) containing protease and phosphatase inhibitors (KeyGEN, Nanjing, China). Equal amounts of total protein (30 μg of protein per lane) were loaded onto gels for electrophoresis and then transferred onto PVDF membranes (Millipore, Billerica, USA), followed by incubation in blocking buffer (25 mM Tris, pH 7.4, 0.15 M NaCl, 0.1% Tween-20, 5% nonfat milk) for 1 h at RT. The membranes were incubated with primary antibodies against α-smooth muscle actin (α-SMA) (Proteintech, Chicago, USA), IL-25 (R&D, Minneapolis, USA), total ERK1/2, phospho-ERK1/2, total AKT, phospho-AKT, phospho-B-Raf (Cell Signaling Technology, Boston, USA) or GADPH (Proteintech, Chicago, USA) at 4 °C overnight. After washing, the membranes were incubated with HRP-conjugated secondary antibodies for 1 h at RT. Proteins were visualized with an ECL kit (Advansta, CA, USA). GADPH was used as a loading control. The intensity of Western blotting bands was analyzed with Quantity One v4.6.2 software.

### Cell migration assays

Cell migration assays were performed as previously described [35]. Briefly, the upper wells of transwells with 8.0-μm pore polycarbonate membrane inserts in a 24-well plate (Corning, NY, USA) were filled with 100 μl of serum-free medium containing 5 × 10^4^ LX-2 cells, and the lower wells contained IL-25 (20 ng/ml) in 600 μl of serum-free medium. The plate was incubated for 12 h at 37 °C. Migrated cells adhering to the undersides of inserts were fixed with 100% methanol for 30 min and then stained with 0.1% crystal violet (Leagene, Beijing, China) for 15 min. Migrated cells were counted by enumerating the number of stained cells in five independent fields for each experiment under a light microscope (Leica, Wetzlar, Germany).

### Statistical analysis

All data are presented as the mean ± SEM. Data were analyzed by performing independent Student’s *t*-tests and ANOVA followed by Bonferroni’s *post-hoc* multiple comparisons test using SPSS software for Windows (version 16.0; SPSS, Inc., IL, USA). A *P* value <0.05 was considered statistically significant.

## Results

### *Cs*LysoPLA upregulated IL-25 expression in the RAW264.7 macrophage cell line

**Fig. 1:**
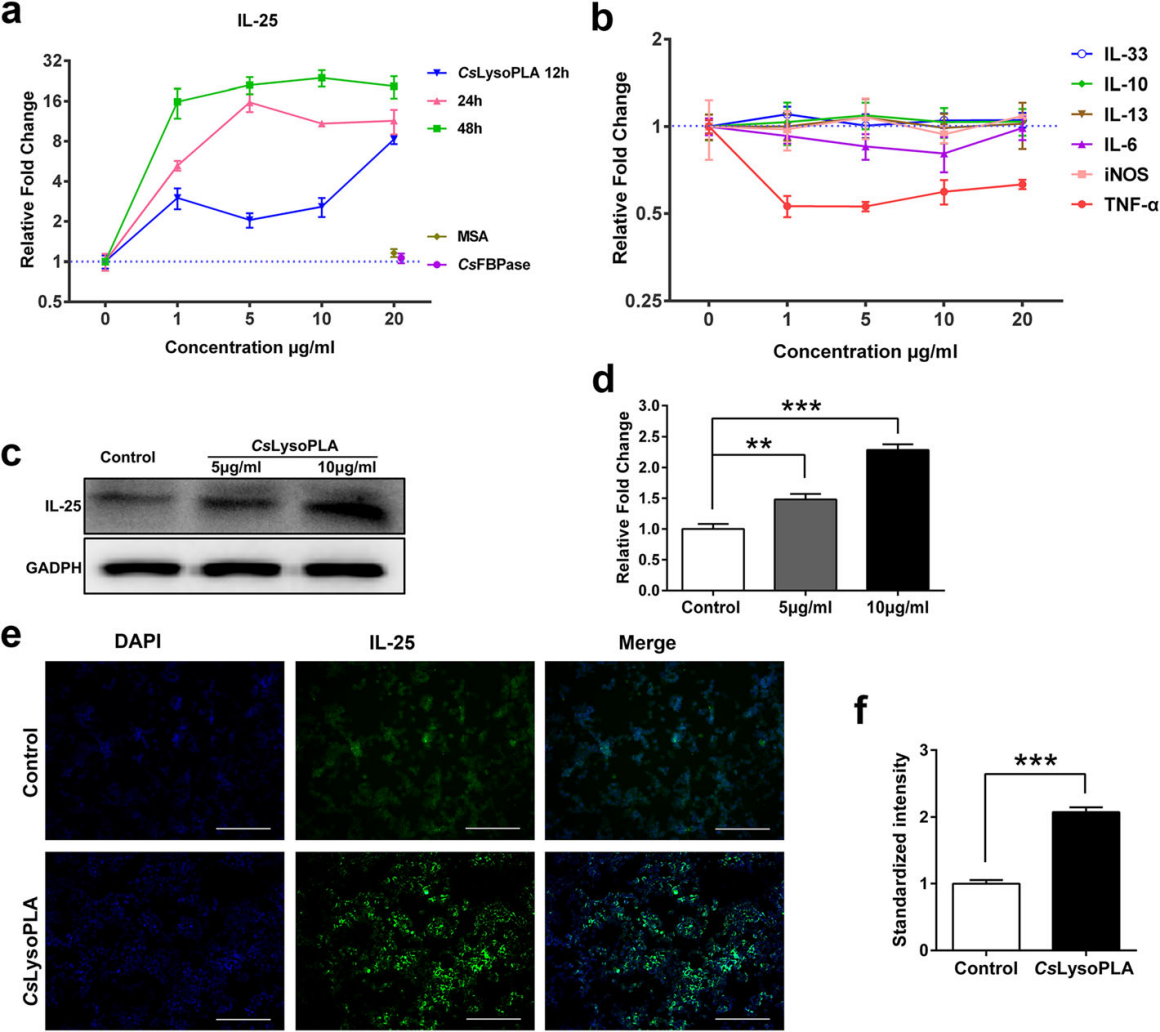
IL-25 is highly expressed in *Cs*LysoPLA-stimulated RAW264.7 cells. **a** Quantitative real-time PCR analysis of IL-25 in RAW264.7 cells treated with *Cs*LysoPLA (1, 5, 10 and 20 μg/ml) and PBS (0 μg/ml, negative control) for 12, 24 and 48 h. *Cs*FBPase (20 μg/ml) and MSA (20 μg/ml) were used as control proteins. Data are shown as mean ± SEM. **b** Quantitative real-time PCR analysis of TNF-α, iNOS, IL-6, IL-13, IL-10 and IL-33 in RAW264.7 cells treated with *Cs*LysoPLA (1, 5, 10 and 20 μg/ml) and PBS (0 μg/ml, negative control) for 24 h. **c** Western blot analysis of IL-25 in RAW264.7 cells treated with *Cs*LysoPLA (10 μg/ml) and the equal volume of PBS for 24 h. GADPH was used as a loading control. **d** Quantification of western blot data in Fig. 1c. Data are shown as mean ± SEM. ** *P* < 0.01, ***P < 0.001. **e** Immunofluorescence staining analysis of IL-25 (*green*) in RAW264.7 cells treated with *Cs*LysoPLA (10 μg/ml) and the equal volume of PBS for 24 h. Nuclei were stained with DAPI (blue). Original magnification ×100. *Scale-bars*: 200 μm. **f** Quantification of immunofluorescence data in Fig. 1e. Data are shown as mean ± SEM. ***P < 0.001

*Cs*LysoPLA stimulation increased IL-25 transcription levels in a time-dependent manner in RAW264.7 cells (*F*_(238)_ = 27.805, *P* < 0.0001) (Fig. 1a). To confirm the specificity of this *Cs*LysoPLA-induced IL-25 transcription, *Cs*FBPase, a member of the *C. sinensis* excretory/secretory proteins (*Cs*ESPs), and MSA were applied as controls. Unlike *Cs*LysoPLA, incubation with *Cs*FBPase or MSA did not elicit significant changes in IL-25 mRNA levels (Fig. 1a). Furthermore, the levels of other cytokines associated with macrophage function, such as TNF-α, iNOS, IL-6, IL-4, IL-13, IL-10 and IL-33, did not change significantly in RAW264.7 cells (Fig. 1b). Western blotting also revealed an increase in IL-25 protein expression compared with the expression in PBS-treated RAW264.7 cells (Fig. 1c, d). Similarly, stronger green fluorescence emitted by an anti-IL-25 monoclonal antibody was observed in RAW264.7 cells incubated with *Cs*LysoPLA compared with the PBS group (Fig. 1e, f).

### *Cs*LysoPLA stimulated IL-25 expression in RAW264.7 cells *via* the PKA-dependent B-Raf-ERK1/2 signaling pathway

**Fig. 2:**
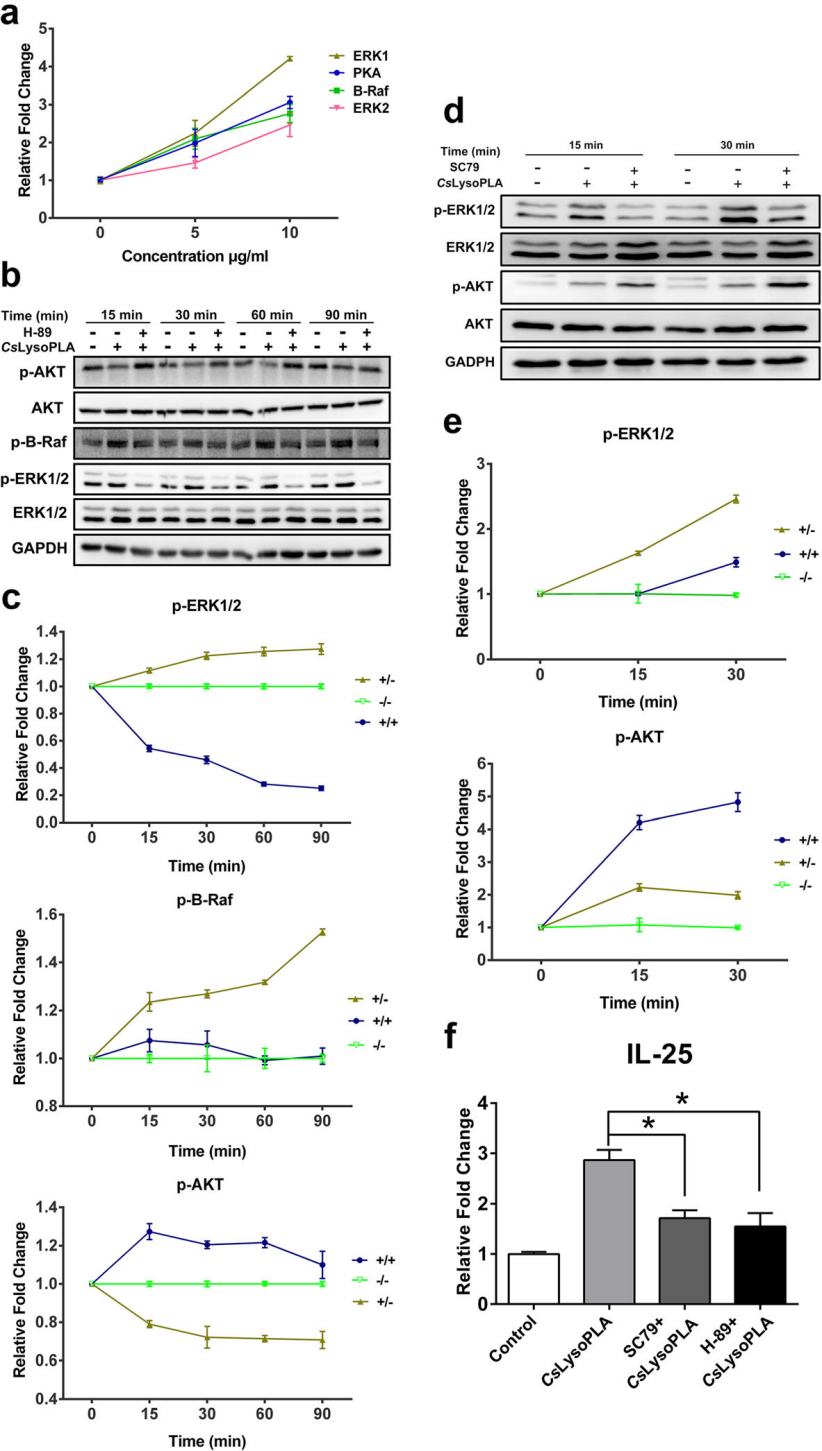
*Cs*LysoPLA facilitates IL-25 expression in RAW264.7 cells *via* PKA-dependent B-Raf-ERK1/2 pathway. **a** RAW264.7 cells were stimulated with *Cs*LysoPLA (5, 10 μg/ml) for 24 h. Relative expressions of PKA, B-Raf, and ERK1/2 genes were examined by Quantitative real-time PCR. **b** RAW264.7 cells were stimulated with *Cs*LysoPLA (10 μg/ml) in the absence or presence H-89 (20 μM) for 15, 30, 60 and 90 min. The protein levels of phospho-B-Raf, total ERK1/2, phospho-ERK1/2, total AKT, phospho-AKT were detected by western blotting with their respective antibodies. GADPH was used as a loading control. **c** Quantification of western blot data in (**b)**. “−/−” PBS control, “+/−” cells treated with *Cs*LysoPLA and “+/+” cells treated with *Cs*LysoPLA and H-89. **d** RAW264.7 cells were stimulated with *Cs*LysoPLA (10 μg/ml) with or without SC79 (4 μg/ml) for 15 and 30 min. The protein levels of total ERK1/2, phospho-ERK1/2, total AKT, phospho-AKT were detected by western blotting with their respective antibodies. PBS was used as negative control. **e** Quantification of western blot data in (**d)**. “−/−” PBS control, “+/−” cells treated with *Cs*LysoPLA and “+/+” cells treated with *Cs*LysoPLA and SC79. **f** RAW264.7 cells were stimulated by *Cs*LysoPLA (10 μg/ml) in the presence of H-89 (20 μM) or SC79 (4 μg/ml) for 12 h. The level of IL-25 mRNA was analyzed by quantitative real-time PCR. Data are shown as mean ± SEM. *P < 0.05

After 24 h, increased mRNA levels of protein kinase A (PKA) (*F*_(2,6)_ = 19.815, *P* = 0.002), B-Raf (*F*_(2,6)_ = 17.593, *P* = 0.003), extracellular signal-regulated kinase 1 (ERK1) (*F*_(2,6)_ = 61.151, *P* < 0.0001) and extracellular signal-regulated kinase 2 (ERK2) (*F*_(2,6)_ = 14.275, *P* = 0.005) were detected in *Cs*LysoPLA-stimulated RAW264.7 cells (Fig. 2a). Western blotting showed that *Cs*LysoPLA induced both B-Raf and downstream ERK1/2 phosphorylation 15 min after stimulation, and phosphorylation gradually increased until 90 min after stimulation (Fig. 2b, c). By contrast, *Cs*LysoPLA inhibited AKT phosphorylation. The PKA inhibitor H-89 attenuated B-Raf and ERK1/2 phosphorylation from 15 min to 90 min (Fig. 2b, c). The AKT activator SC79 also reduced the levels of phosphorylated ERK1/2 in RAW264.7 cells (Fig. 2d, e). Moreover, both H-89 (*t*_(4)_ = 3.933, *P* = 0.017) and SC79 (*t*_(4)_ = 4.480, *P* = 0.011) inhibited IL-25 expression induced by *Cs*LysoPLA (Fig. 2f).

### IL-25-induced activation and migration of LX-2 cells

**Fig. 3:**
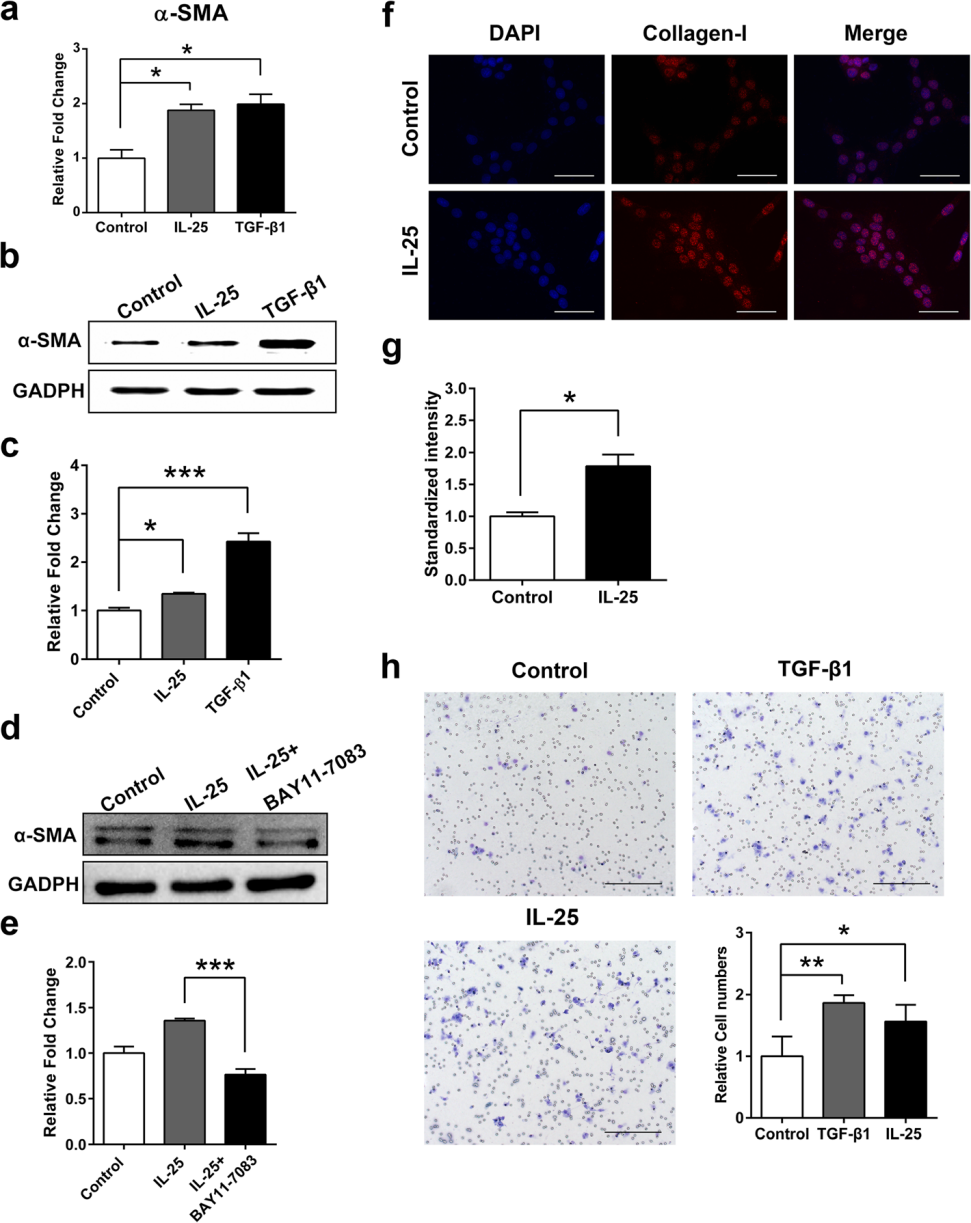
IL-25 facilitates LX-2 cells activation and migration. **a** LX-2 cells were stimulated with IL-25 (20 ng/ml) and TGF-β1 (5 ng/ml) for 24 h. Relative α-SMA expression was examined by quantitative real-time PCR. **b** LX-2 cells were stimulated with IL-25 (20 ng/ml) and TGF-β1 (5 ng/ml) for 24 h. α-SMA protein expression was detected by western blotting. GADPH was used as loading control. **c** Quantification of western blot data in (**b)**. **d** LX-2 cells were stimulated with IL-25 (20 ng/ml) in the absence or presence of BAY 11–7083 (0.1 μg/ml) for 24 h. α-SMA protein expression was detected by western blotting. GADPH was used as loading control. **e** Quantification of western blot data in (**d)**. **f** LX-2 cells were stimulated with IL-25 (20 ng/ml) for 24 h. Collagen-I protein expression was determined by immunofluorescence staining using Collagen-I antibody (*red*). Nuclei were stained with DAPI (*blue*). Original magnification ×200. **g** Quantification of immunofluorescence data in Fig. 3f. **h** LX-2 cells were stimulated with IL-25 (20 ng/ml) for 24 h. The migration was analyzed using a Transwell plate. Light microscopy was used to collect images of migrated cells. Original magnification ×100. The number of migration cells was counted as a mean of five independent fields for each experiment. PBS was used as negative control and TGF-β1 as the positive control. Data are shown as mean ± SEM. *P < 0.05, **P < 0.01, *** *P* < 0.001. *Scale-bars*: **f**, 100 μm; **h**, 200 μm

Both α-SMA and Collagen-I are markers of activated HSCs [19]. α-SMA mRNA levels in LX-2 cells were significantly upregulated (*t*_(4)_ = 4.634, *P* = 0.010) following stimulation with IL-25 for 24 h (Fig. 3a), and α-SMA protein levels exhibited the same change (Fig. 3b, c). Furthermore, LX-2 cells were pretreated with an NF-κB inhibitor (BAY 11–7083) before stimulation with IL-25. BAY 11–7083 blockade resulted in a reduction in α-SMA protein expression compared with IL-25 treatment (Fig. 3d, e). Red fluorescence from a Cy3-conjugated anti-collagen-I monoclonal antibody was markedly stronger in IL-25-treated LX-2 cells compared with cells that underwent PBS treatment (Fig. 3f, g). In addition, much greater amounts of LX-2 cells migrated to the lower well following IL-25 treatment compared with PBS-treated cells (*t*_(4)_ = 2.984, *P* = 0.017) (Fig. 3h, i).

## Discussion

Liver fibrosis caused by *C. sinensis* infection affects patient quality of life, but the underlying mechanisms have yet to be clarified. Liver fibrosis is a well-known repair response during liver injury, and HSCs and many cytokines take part in its progression [19–21]. In the current study, treatment with *Cs*LysoPLA induced IL-25 expression in RAW264.7 cells. PKA, B-Raf, and ERK1/2 mRNA levels in *Cs*LysoPLA-stimulated RAW264.7 cells increased. *Cs*LysoPLA induced the phosphorylation of both B-Raf and ERK1/2, whereas the PKA inhibitor H-89 attenuated B-Raf and ERK1/2 phosphorylation, confirming the role of *Cs*LysoPLA. The AKT activator SC79 reduced the levels of phosphorylated ERK1/2 in RAW264.7 cells. Both H-89 and SC79 inhibited IL-25 upregulation induced by *Cs*LysoPLA. In addition, IL-25 upregulated the expression of α-SMA and Collagen-I in LX-2 cells and promoted cell migration.

The host immune response to *C. sinensis* infection tends to be Th2-dominant [24, 25]. IL-25 is a Th2 cytokine regulator, and previous reports revealed an association between IL-25 and pulmonary disorders such as pulmonary fibrosis and airway remodeling [14, 36]. According to a previous study investigating a rat model of particle-induced airway inflammation, macrophages are potential sources of IL-25 [9]. Additionally, *Cs*LysoPLA was proposed to play a role in *C. sinensis*-induced liver fibrosis. Therefore, we stimulated RAW264.7 cells with *Cs*LysoPLA and observed that *Cs*LysoPLA significantly promoted the expression of IL-25 but not TNF-α, iNOS, IL-6, IL-4, IL-13, IL-10 or IL-33, which mediate macrophage functions [37, 38]. Thus, *Cs*LysoPLA may interfere with macrophage function by upregulating IL-25 expression. An excretory/secretory protein from *C. sinensis*, *Cs*FBPase, did not elevate IL-25 expression in RAW264.7 cells when applied as a control, which suggests a specific function for *Cs*LysoPLA in RAW264.7 cells.

Rat lysophospholipase removes palmitate from G_α_ subunits, accelerating the cycling of G_α_ subunits between palmitoylation and depalmitoylation and resulting in increased G protein signaling efficacy [39]. The ERK signaling cascade plays an important role in regulating gene expression, cell proliferation and differentiation, and apoptosis [40–42]. ERK1/2 activation is modulated by G_α_ *via* the cAMP/PKA signaling cascade, which ultimately activates the B-Raf-MEK-ERK module [43–45]. In addition, the AKT and ERK pathways undergo negative crosstalk to induce AKT-mediated ERK signaling pathway inactivation [46–48]. In the present study, we observed PKA, B-Raf and ERK1/2 activation and AKT inhibition. Blocking PKA and activating AKT with the chemical inhibitors H-89 and SC79, respectively, further confirmed the signaling pathways involved in *Cs*LysoPLA-induced IL-25 expression. Enhanced AKT activation, inhibited ERK1/2 excitation and attenuated *Cs*LysoPLA-induced IL-25 overexpression were present in RAW264.7 cells. These results suggested that the ERK1/2 signaling pathway is involved in *Cs*LysoPLA-induced IL-25 elevation in macrophages; additionally, there may be other transcriptional mediators contributing to IL-25 production. In summary, *Cs*LysoPLA-mediated IL-25 production is partially dependent on the PKA-dependent B-Raf/ERK1/2 pathway.

Macrophages play a major functional role in liver fibrosis. Both macrophage depletion in *Cd11b-DTR* transgenic mice and macrophage blockade in mice *via* liposomal clodronate injection in response to CCL4 resulted in prominently reduced HSC activation and numbers as well as attenuated fibrosis [49, 50]. To our knowledge, macrophages take part in the development of liver fibrosis by secreting a diverse range of cytokines, chemokines and other soluble regulators that directly act on HSCs [23].

HSC activation represents a pivotal event in liver fibrosis [51]. Activated HSCs convert to a myofibroblast-like phenotype, upregulate mesenchymal cell markers such as α-SMA and Collagen-I, and migrate to sites of damage [19, 23]. IL-25 significantly increases collagen secretion by normal human lung fibroblasts [14]. In the present study, we first investigated the direct interaction between IL-25 and HSCs. IL-25 enhanced the expression of α-SMA and Collagen-I and promoted the migration of LX-2 cells. Both of these effects likely result in the secretion and accumulation of excessive ECM proteins and facilitate the pathogenesis of liver fibrosis [51]. Thus, the present findings suggest IL-25 may be a profibrotic cytokine that regulates fibrogenesis by directly activating HSCs and promoting their migration. Recent studies indicate NF-κB is essential for IL-25-mediated inflammation and hyper-responsiveness [52, 53]. We observed inhibited α-SMA expression in LX-2 cells following the blockade of NF-κB with a chemical inhibitor (BAY 11–7083), suggesting that IL-25 may stimulate the expression of profibrotic genes in HSCs *via* NF-κB signaling pathway activation. The precise mechanism underlying IL-25 function requires further investigation.

## Conclusions

**Fig. 4:**
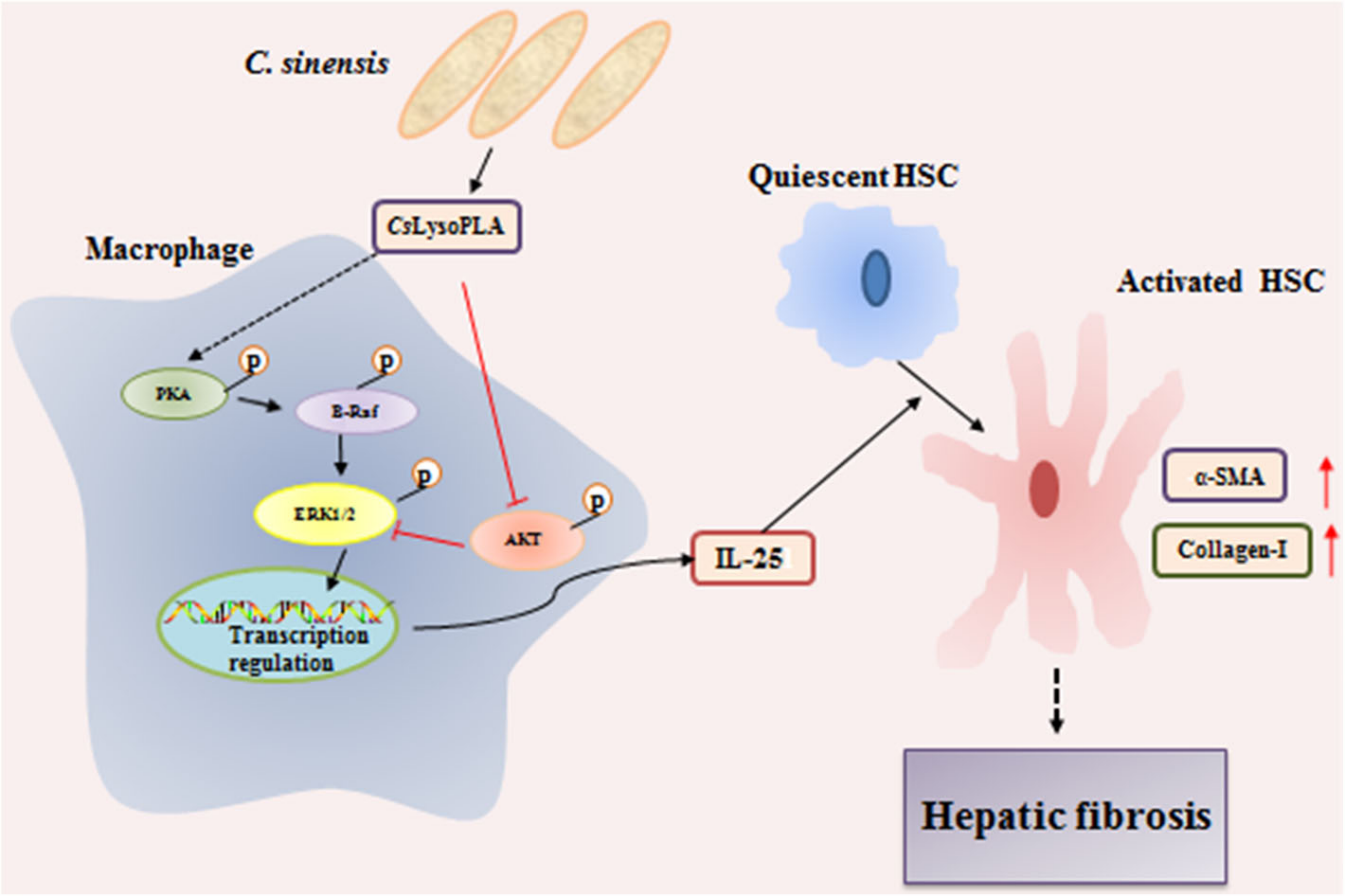
Schematic chart showing the potential role of *Cs*LysoPLA in hepatic fibrosis. *Cs*LysoPLA activates PKA, B-Raf and ERK1/2, and inhibits AKT phosphorylation, upregulating IL-25 in macrophages. IL-25 enhances the expression of α-SMA and Collagen-I in LX-2 cells, promoting hepatic fibrosis

Our previous work showed that IL-25 is significantly elevated in the serum of *C. sinensis*-infected mice, and this trend correlated with the degree of liver fibrosis during infection. Based on these results, we speculate that *Cs*LysoPLA infiltrates blood capillaries broken following mechanical damage and chemical injury induced by the adult worm and its excretory/secretory products, then activates HSCs by upregulating IL-25 in macrophages through the PKA-dependent B-Raf/ERK1/2 pathway, thus promoting hepatic fibrosis during infection (Fig. 4). This hypothesis needs further verification in vivo in future studies. Nonetheless, our work may provide valuable information for the development of liver fibrosis therapies.

## Abbreviations

Collagen-I: Collagen type I
*Cs*ESPs: *C. sinensis* excretory/secretory proteins
*Cs*FBPase: *C. sinensis* Fructose-1, 6-bisphosphatase
*Cs*LysoPLA: *C. sinensis* lysophospholipase A
DAPI: 4′,6-diamidino-2-phenylindole
DMEM: Dulbecco’s modified Eagle’s medium
ECM: Extracellular matrix
FBS: Fetal bovine serum
HSCs: Hepatic stellate cells
MSA: Mouse serum albumin
PBS: Phosphate-buffered saline
Th2: Helper T cells.
α-SMA: α-smooth muscle actin
